# Whole Exome Sequencing with Comprehensive Gene Set Analysis Identified a Biparental-Origin Homozygous c.509G>A Mutation in *PPIB* Gene Clustered in Two Taiwanese Families Exhibiting Fetal Skeletal Dysplasia during Prenatal Ultrasound

**DOI:** 10.3390/diagnostics10050286

**Published:** 2020-05-07

**Authors:** Ting-Yu Chang, I-Fang Chung, Wan-Ju Wu, Shun-Ping Chang, Wen-Hsiang Lin, Norman A. Ginsberg, Gwo-Chin Ma, Ming Chen

**Affiliations:** 1 Department of Genomic Medicine and Center for Medical Genetics, Changhua Christian Hospital, Changhua 50046, Taiwan; 182451@cch.org.tw (T.-Y.C.); crystalwu835@gmail.com (W.-J.W.); 70914@cch.org.tw (S.-P.C.); 397620cch@gmail.com (W.-H.L.); 2 Research Department, Changhua Christian Hospital, Changhua 50006, Taiwan; 3 Department of Genomic Science and Technology, Changhua Christian Hospital Healthcare System, Changhua 50046, Taiwan; 4 Department of Bioscience Technology, Chung Yuan Christian University, Taoyuan 32023, Taiwan; 5 Institute of Biomedical informatics, National Yang-Ming University, Taipei 11221, Taiwan; ifchung@ym.edu.tw; 6 Department of Obstetrics and Gynecology, Changhua Christian Hospital, Changhua 50006, Taiwan; 7 Ph.D. programs in Translational Medicine, National Chung Hsing University, Taichung 40227, Taiwan; 8 Department of Obstetrics and Gynecology, Feinberg School of Medicine, Northwestern University Medical Center, Chicago, IL 60611, USA; cvsguy1@aol.com; 9 Department of Biomedical Engineering, Chung Yuan Christian University, Taoyuan 32023, Taiwan; 10 Department of Medical Laboratory Science and Biotechnology, Central Taiwan University of Science and Technology, Taichung 40601, Taiwan; 11 Department of Medical Genetics, National Taiwan University Hospital, Taipei 10041, Taiwan; 12 Department of Obstetrics and Gynecology, College of Medicine, National Taiwan University, Taipei 10041, Taiwan; 13 Department of Biomedical Science, Dayeh University, Changhua 51591, Taiwan; 14 Department of Medical Science, National Tsing Hua University, Hsinchu 30013, Taiwan

**Keywords:** WES, fetal diagnosis, skeletal dysplasia, *PPIB*, trio analysis, osteogenesis imperfecta

## Abstract

Skeletal dysplasia (SD) is a complex group of bone and cartilage disorders often detectable by fetal ultrasound, but the definitive diagnosis remains challenging because the phenotypes are highly variable and often overlap among different disorders. The molecular mechanisms underlying this condition are also diverse. Hundreds of genes are involved in the pathogenesis of SD, but most of them are yet to be elucidated, rendering genotyping almost infeasible except those most common such as fibroblast growth factor receptor 3 (*FGFR3*), collagen type I alpha 1 chain (*COL1A1*), collagen type I alpha 2 chain (*COL1A2*), diastrophic dysplasia sulfate transporter (*DTDST*), and SRY-box 9 (*SOX9*). Here, we report the use of trio-based whole exome sequencing (trio-WES) with comprehensive gene set analysis in two Taiwanese non-consanguineous families with fetal SD at autopsy. A biparental-origin homozygous c.509G>A(p.G170D) mutation in peptidylprolyl isomerase B (*PPIB*) gene was identified. The results support a diagnosis of a rare form of autosomal recessive SD, osteogenesis imperfecta type IX (OI IX), and confirm that the use of a trio-WES study is helpful to uncover a genetic explanation for observed fetal anomalies (e.g., SD), especially in cases suggesting autosomal recessive inheritance. Moreover, the finding of an identical *PPIB* mutation in two non-consanguineous families highlights the possibility of the founder effect, which deserves future investigations in the Taiwanese population.

## 1. Introduction

Skeletal dysplasia (SD), or osteochondrodysplasias, is a heterogeneous group of chondro-osseous disorders that comprises more than 450 distinctive disease entities [1]. SD is categorized into three groups: osteodysplasia, chondrodysplasias, and dysostosis. Osteodysplasia is characterized by abnormalities in bone, leading to abnormal bone density and mineralization; chondrodysplasia is related abnormalities in cartilage, leading to short stature caused by defective linear growth; and dysostosis is an anomaly of a single bone or a group of bones that affects certain skeletal elements [2]. Recently, the distinction between the groups has blurred because of overlapping features and increasing intricacy [3]. SD features such as short/bowing/fractured long bones, hypo-ossification of long bones/calvarium/vertebrae, dysmorphic scapula/rib/calvarium/long bones/vertebrae, limb deformity including club foot (equinovarus), polydactyly/clinodactyly, clenched fists, joint contractures (arthrogryposis), absence or deficiency of certain bony component, small chest, etc. are typically detected in prenatal ultrasound. However, experienced sonographers can only confidently diagnose a few conditions with featured sonographic findings such as achondroplasia, achondrogenesis, asphyxiating thoracic dysplasia (Jeune syndrome), atelosteogenesis, thanatophoric dysplasia, camptomelic dysplasia, short rib-polydactyly syndrome, diastrophic dysplasia, Ellis van Creveld syndrome, Adams–Oliver syndrome, arthrogryposis multiplex congenita, and osteogenesis imperfecta (OI) [4]. This poses a dilemma for families and physicians in genetic counseling because some SD diseases are lethal, despite the fact that in most conditions, intelligence is not affected [1]. Molecular analysis has been an acceptable approach for the genetic diagnosis of SD even though this is challenging because too many genes are involved and in many conditions, the causative genes remain unknown. Currently, more than 300 genes are associated with SD [5,6,7,8,9]. Sanger sequencing has been regularly used for genotyping of some common SD genes such as fibroblast growth factor receptor 3 (*FGFR3*) (thanatophoric dysplasia and achondroplasia), collagen type I alpha 1 chain (*COL1A1*) and collagen type I alpha 2 chain (*COL1A2*) (OI), diastrophic dysplasia sulfate transporter (*DTDST*) (diastrophic dysplasia and atelosteogenesis), and SRY-box 9 (*SOX9*) (camptomelic dysplasia), but this technique becomes costly and time-consuming when a great number of genes are tested before reaching a definite molecular diagnosis. Whole exome sequencing (WES) sequences all of the protein-coding regions of genes in a genome [10] and is therefore a feasible tool to elucidate the genes/mutations underlying such complex conditions. Here, we present two unrelated Taiwanese families with fetal SD who received an abortus-mother-father trio-based WES (trio-WES) study with comprehensive gene set analysis at fetal autopsy, and the etiologies were identified.

## 2. Materials and Methods 

### 2.1. Patients

This study was approved by the Institutional Review Board of Changhua Christian Hospital, Taiwan (Project No.:190116; approval date: 06 June 2019). Two pregnant women whose fetuses (patients 1 and 2) were suspected to be affected with SD according to the prenatal ultrasound were enrolled in this study. Informed consents were obtained. The clinical information and prenatal findings are described below.

#### 2.1.1. Patient 1

**Figure 1:**
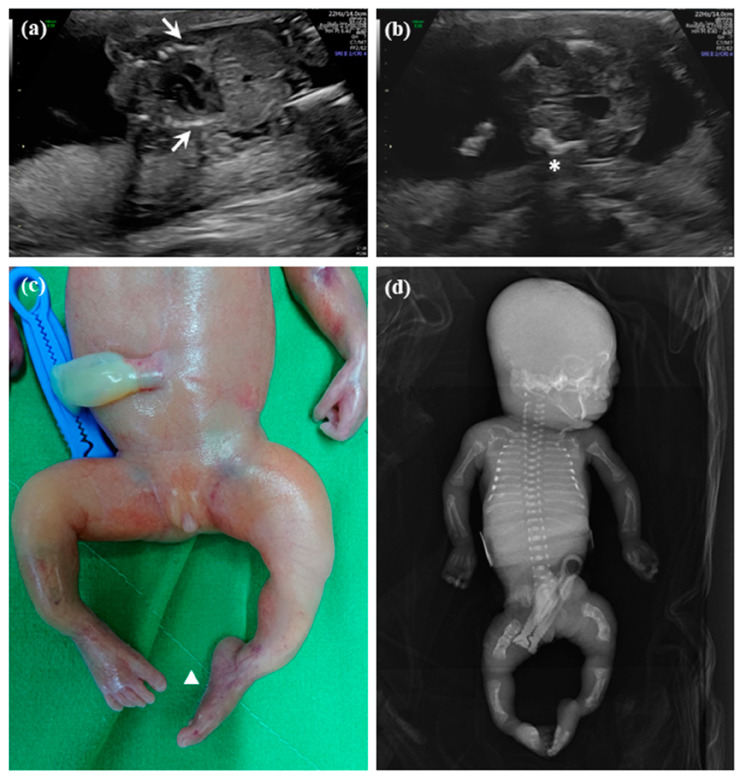
Prenatal ultrasonography of Patient 1 (a female fetus) with osteogenesis imperfecta type IX (OI IX), a kind of skeletal dysplasia (SD), in Family 1 at gestation age (GA) = 19 weeks and 5 days showed (**a**) bell-shape chest (arrow) and (**b**) bowing of femur (star). The appearance of the abortus showed (**c**) talipes equinovarus (triangle). Postmortem whole-body x-film showed (**d**) thinning of ribs, and bowing of femurs, tibias, and fibulas.

A 36-year-old female, G2P0SA1, visited our hospital because of shortened long bones onset at the second trimester. She denied consanguineous marriage and underwent regular antenatal care in local clinics. Noninvasive prenatal testing (NIPT) showed low risk for trisomy 13, 18, and 21. At the first visit at gestational age (GA) = 19 weeks and 5 days, the fetal anomaly scan revealed a structurally normal female fetus except a bell-shaped chest with rib fracture (Figure 1a) and shortened long bones over the four limbs (around GA = 17 weeks by estimation), of which the lower limbs were worse than the upper ones with bowed femurs at both lower limbs (Figure 1b). The skull showed hypomineralization and plagiocephly (not shown here). No other structural anomalies were found. The GA dating was determined by serial ultrasound measurement during the first trimester by crown rump length (CRL), which follows the norms of obstetrics. After non-directive counselling, the pregnant woman chose termination of pregnancy (TOP) at GA = 21 weeks and 3 days. Fetal autopsy as well as genetic survey were conducted to better delineate the etiology of this case. A female abortus was delivered vaginally, which showed talipes equinovarus (Figure 1c). Postmodern whole-body X-ray demonstrated widening of the growth plate, accompanied by spreading, cupping, and irregularity or fraying of the metaphysis of the growing ends at both tibia and fibula. The ribs were thin and the femoral, tibia, and fibular bones were bowed (Figure 1d). Given the presence of a coarse trabecular pattern with generalized rarefaction, the radiologist suspected it was a case of rickets (but was less likely from our prenatal experience). Cytogenetic analysis showed a normal female karyotype 46,XX. Chromosome microarray analysis (CMA) by the oligonucleotide 8×60K CytoScan^®^ gene chip (Agilent customer design ID 040427, Changhua Christian Hospital, Changhua, Taiwan) revealed no abnormality (arr(1-22,X)×2). Sanger sequencing for the most common SD genes including *FGFR3*, *COL1A1*, *COL1A2*, *DTDST*, and *SOX9* were all negative. To uncover the underlying genetic defect of this case, DNA from the abortus (Patient 1), mother, and father were gathered for the trio-WES study. 

#### 2.1.2. Patient 2

**Figure 2:**
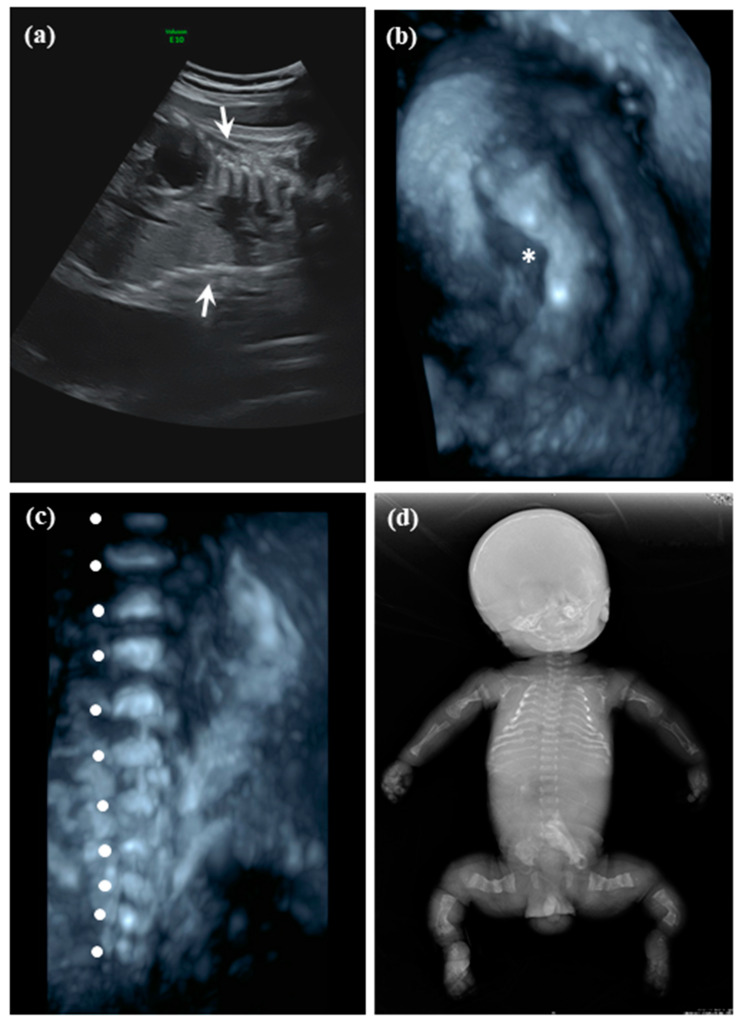
Prenatal ultrasonography of Patient 2 (a male fetus) with OI IX in Family 2 at GA = 34 weeks and 1 day showed (**a**) small and collapsed thoracic cage (arrow), (**b**) bowing of femoral bone (star), and (**c**) platyspondyly of spine (filled circle). Postmortem whole-body x-film showed (**d**) ribbon like ribs, multiple bowing and fractures of general gracile bones, and pseudoarthrosis of long bones.

A 33-year-old female, gravida 2 para 1, was referred to our hospital for further evaluation in the third trimester due to fetal bony anomalies. She denied consanguineous marriage and any relevant history over her first healthy child. The results of all antenatal care including Down syndrome screening were uneventful until the third trimester, when OI was suspected. At visit, she was at GA = 34 weeks and 1 day (determined by examining previous records of antenatal visits regarding serial ultrasound measurements and last menstrual period). The anatomic ultrasound screening showed small thoracic cage (Figure 2a), micromelia (approximately GA = 22–23 weeks by estimation) with bowing of femoral bones (Figure 2b), and platyspondyly of spine (Figure 2c). In addition, clenched fists, club feet, and demineralization of the skull were also noted (not shown here). Due to poor prognosis, she opted for late TOP at GA = 35 weeks and 1 day after non-directive genetic counselling. The procedure is based on the principle for late TOP proposed by the Taiwan Association of Obstetrics and Gynecology. Autopsy, whole-body X-ray examination and genetic analyses were conducted on the abortus. The postmortem roentgenography demonstrated general deformed, gracile bones with ribbon-like ribs, bowing, multiple fractures, and pseudoarthrosis of bilateral long bones (Figure 2d). Cytogenetic analysis revealed male karyotype with a normal variant 46,XY,16qh+. CMA revealed no abnormality (arr(1−22)×2,(X,Y)×1). Sanger sequencing for *FGFR3*, *COL1A1*, *COL1A2*, *DTDST*, and *SOX9* were negative. To delineate the possible underlying genetic defect, DNA from the abortus (Patient 2) and both parents were collected for a trio-WES study.

### 2.2. Whole Exome Sequencing (WES) by Next Generation Sequencing (NGS)

Since more than 300 genes are associated with SD and Sanger sequencing for common SD genes (*FGFR3*, *COL1A1*, *COL1A2*, *DTDST*, and *SOX9*) were negative, we utilized trio-WES to screen genetic variations including both abortuses (Patients 1 and 2) and their parents. Genomic DNA was isolaged from 200 µL of peripheral whole blood using Qiagen DNA Blood Mini Kit (Qiagen, Hilden, Germany). The DNA quality was measured based on optical density (OD) using Nano Drop (Thermo Fisher Scientific, MA, USA) to control the protein contamination (OD260/280 > 1.8) and organic solvent contamination (OD260/230 > 1.3.) Then, the double stranded DNA concentration was measured by Qubit dsDNA BR Assay Kit (Thermo Fisher Scientific, MA, USA) to avoid overestimating OD level from the degraded nucleotide. Finally, the integrity of genomic DNA was evaluated by gel electrophoresis using Agilent Tape Station (Agilent, CA, USA) to ensure a sharp signal around 20 kb and no obvious smearing signal was observed below 10 kb.

Purified genomic DNA were subjected to ultrasonic fragmentation by a Covaris S220 sonicator (Covaris, MA, USA) to obtain a DNA fragment size ranging from 200 to 500 bps. Fragmented genomic DNA was ligated with sample specific barcode sequences and a pair of universal tags (Illumina, CA, USA) following polymerase chain reaction (PCR) low cycle (8–12) amplification.

The exonic region of genomic DNA samples were then enriched by hybridization with Agilent SureSelect Clinical Research v2 probes (Agilent, MA, USA) following the manufacturer’s instructions. After washing out non-captured intron/intergenic DNA, the purified exonic DNA were subjected to Illumina NextSeq 500 (Illumina, CA, USA) for next generation sequencing (NGS) with a 2 × 150 bp format. The sequencing amount for each sample was estimated as 10–12 giga base pairs and the average coverage depth of captured region was more than 50 folds.

### 2.3. Exome Variation Analysis

**Figure 3:**
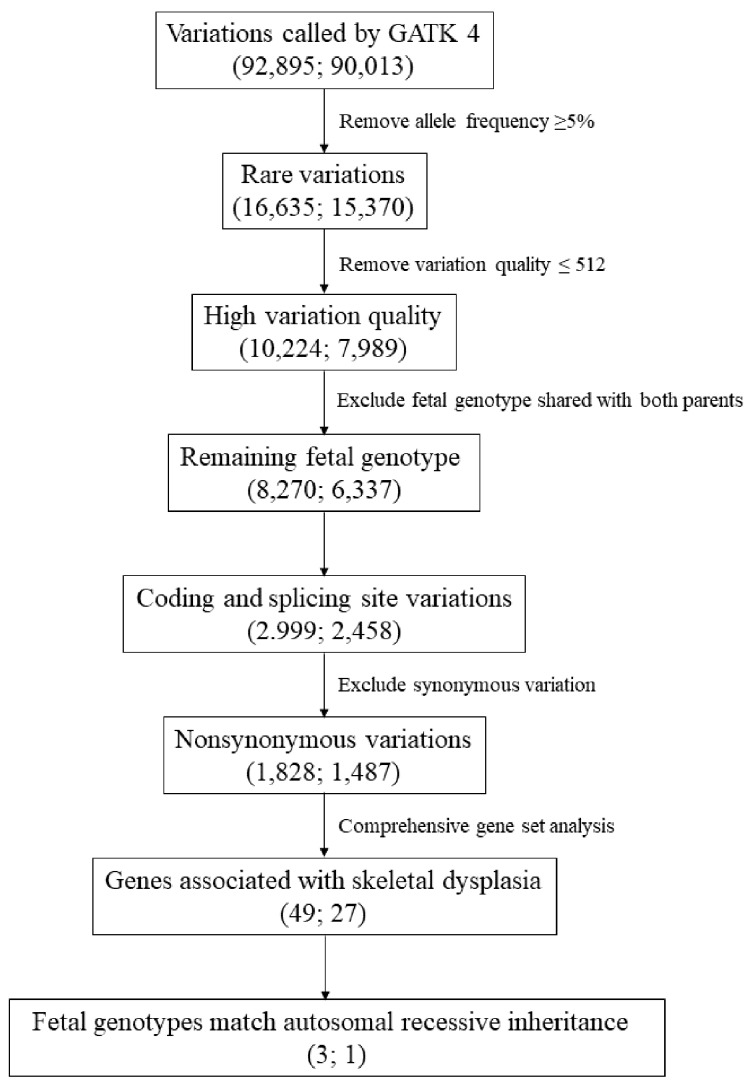
The flow chart of the analysis pipeline of the trio-based whole exome sequencing (trio-WES) data. The numbers of variations selected by each analysis step for Family 1 (left) and Family 2 (right) are indicated in round brackets.

The NextSeq sequencing data were converted into the gzipped fasta format and followed the Genome Analysis Toolkit (GATK) best practice proposed by the Broad Institute. Briefly, the raw fasta sequencing result first depleted the sequencing adapter sequence and aligned to the hg38 (GRCh38.p12) human genome. Mapped results with duplicated reads were marked and the base qualities were recalibrated according to the GATK best practice. The variations were called by a haplotype caller implemented in GATK 4 (version 4.1.4.1), and the results of individual vcf files were merged by a setting of the trio model (proband–mother–father.). The joint vcf file was annotated utilizing resources from University of California, Santa Cruz (UCSC), Ensembl, dbNSFP35a, ClinVar, Variant Effect Predictor (VEP), 1000 genomes project, Exome Aggregation Consortium (ExAC), and The Genome Aggregation Database (gnomAD) to classify the type of variations, population frequencies, and potential impact on protein functions. All variations detected were filtered by a series of criteria including elimination of minor allele frequency (MAF) ≥ 5% in East Asian population from the gnomAD project, elimination of variation calling quality ≤ 512 by the GATK haplotype caller, elimination of genotypes shared with both parents, retention of variations on coding and splicing region, and elimination of synonymous variations (Figure 3). A comprehensive gene set that comprised almost all the reported 317 SD genes [5,6,7,8,9] was also included in our WES analysis pipeline before performing a genome-wide analysis for the identification of novel genes involved in the etiology of SD (Figure 3).

### 2.4. In Silico Analysis

The effect of individual variation on protein function was in silico predicted by programs including Sorting Intolerant From Tolerant (SIFT) (http://sift.bii.a-star.edu.sg/) [11], PolyPhen-2 (http://genetics.bwh.harvard.edu/pph2/) [12], LRT (http://www.genetics.wustl.edu/jflab/lrt_query.html) [13], MutationTaster (http://www.mutationtaster.org/) [14], Mutationassessor (http://mutationassessor.org/r3/) [15], FATHMM (http://fathmm.biocompute.org.uk/) [16], PROVEAN (http://provean.jcvi.org/index.php) [17], MetaSVM (https://sites.google.com/site/jpopgen/dbNSFP [18], MetaLR (https://sites.google.com/site/jpopgen/dbNSFP) [19], and FATHMM-MKL (http://fathmm.biocompute.org.uk/fathmmMKL.htm) [20].

### 2.5. Cross-Species Conservation Analysis

The cross-species conservation at detected variations among the corresponding protein (PPIB) of human and 28 other mammal species (including 26 primate species and two rodent species) was examined by comparing the reported sequences in Ensembl (Human: ENSP00000300026, Angola colobus: ENSCANP00000028938, Black snub nosed monkey: ENSRBIP00000005529, Bolivian squirrel monkey: ENSSBOP00000036621, Bonobo: ENSPPAP00000020477, Bushbaby: ENSOGAP00000001726, Capuchin: ENSCCAP00000039294, Chimpanzee: ENSPTRP00000012236, Coquerel’s sifaka: ENSPCOP00000025497, Crab eating macaque: ENSMFAP00000009363, Drill: ENSMLEP00000008030, Gelada: ENSTGEP00000027639, Gibbon: ENSNLEP00000015077, Golden snub nosed monkey: ENSRROP00000023338, Gorilla: ENSGGOP00000010808, Greater bamboo lemur: ENSPSMP00000003491, Ma’s_night_monkey: ENSANAP00000042013, Macaque: ENSMMUP00000055033, Marmoset: ENSCJAP00000009124, Mouse: ENSMUSP00000034947, Mouse lemur: ENSMICP00000020871, Olive baboon: ENSPANP00000014124, Orangutan: ENSPPYP00000007428, Pig tailed macaque: ENSMNEP00000037065, Rat: ENSRNOP00000022828, Sooty mangabey: ENSCATP00000034856, Tarsier: ENSTSYP00000033454, Ugandan red colobus: ENSPTEP00000034533, and Vervet-AGM: ENSCSAP00000017751). Multiple alignment of protein sequences was performed using ClustalW (https://www.genome.jp/tools-bin/clustalw).

## 3. Results

The trio-WES analysis of Patient 1 identified 92,895 variations including 82,816 single nucleotide variations (SNVs) and 10,079 small insertions/deletions (indels). The trio-WES analysis for Patient 2 identified 90,013 variations including 80,183 SNVs and 9830 indels. After filtering by a series of criteria, 49 and 27 rare and potential deleterious variations associated with SD [21] were identified in Patients 1 and 2, respectively (Figure 3). Since both parents of the two affected fetuses were healthy without SD phenotype, an autosomal recessive (AR) inheritance pattern was speculated. In Patient 1, three genetic variations in the SD gene set, which match a known AR inheritance pattern, were found. The fist variation was in exon 31 of WD repeat domain 19 (*WDR19*) (NM_025132.4:c.3416A>G(p.Q1139R), rs75621037), which causes the 1139th amino acid alteration from glutamine to arginine (Table 1). This variation does not locate at any known repetitive sequences or protein domains. Variation impact prediction on protein function showed tolerated/benign/neutral/polymorphism in seven algorithms (SIFT, Polyphen2, Mutation Assessor, FATHMM, PROVEAN, MetaSVM, and MetaLR) and deleterious/damaging/disease causing in three algorithms (LRT, Mutation Taster, and FATHMM-MKL) (Table 2). The MAF of this variation was 0.939% in the Asian population from the gnomAD-exom project (Table 1). The second variation was in exon 49 of centrosomal protein 290 (*CEP290*) (NM_025114.4:c.6806T>C (p.I2269T), rs200090371), which replaces the 2269^th^ amino isoleucine by threonine (Table 1). The MAF in the Asian population was 0.114%. Variation impact prediction showed tolerated/benign/neutral/polymorphism in nine algorithms (SIFT, Polyphen2, LRT, Mutation Taster, Mutation Assessor, FATHMM, PROVEAN, MetaSVM, and MetaLR) and deleterious/damaging/disease causing in only one algorithm (FATHMM-MKL) (Table 2). The third variation was in exon 4 of *PPIB* (NM_000942.5:c.509G>A(p.G170D), rs199606428), which causes the 170^th^ amino acid to change from glycine to aspartic acid (Table 1). The MAF for the Asian population was 0.035% (Table 1). All 10 algorithms predicted a deleterious/damaging/disease causing effect on the protein function (Table 2). 

**Figure 4:**
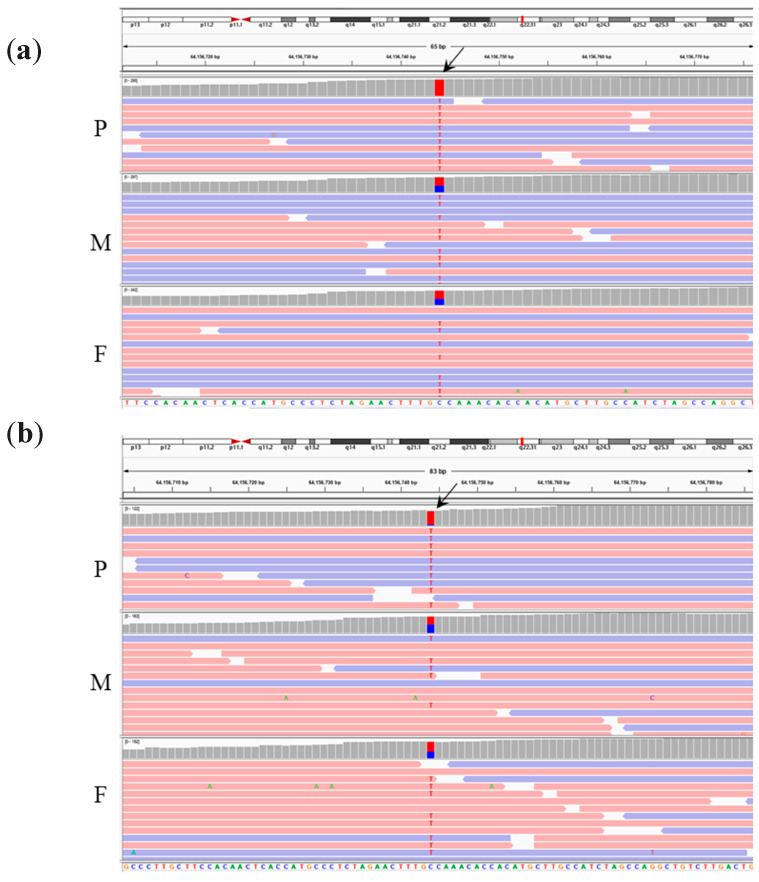
Trio-WES of (**a**) Family 1 and (**b**) Family 2 identified an identical biparental-origin homozygous mutation in *PPIB* (NM_000942.5:c.509G>A(p.G170D), corresponding to chr15 g.64156744C>T, hg38) (arrow) in both patients. P, M, and F indicate patient, mother, and father respectively.

For the second trio-WES analysis of Patient 2, the same analysis pipeline was adopted to identify variations in the SD gene set that possessed an AR inheritance pattern (Figure 3). Only one variation in a homozygous status was identified in *PPIB*. Unexpectedly, the *PPIB* mutation (NM_000942.5:c.509G>A(p.G170D), rs199606428) detected in Patient 2 was identical to that detected in Patient 1. Alignments of the trio-WES captured reads spanning the *PPIB* mutation (NM_000942.5:c.509G>A(p.G170D), rs199606428) in both families are shown in Figure 4.

**Figure 5:**
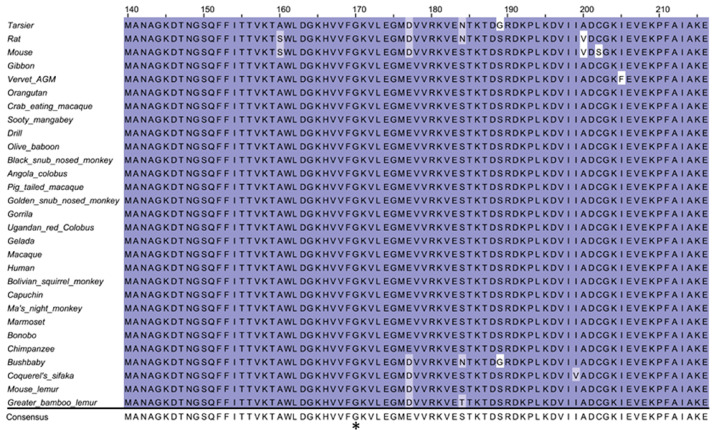
Cross-species conservation analysis of peptidylprolyl isomerase B (*PPIB*) amino acids among human and 28 other mammal species showed a high cross-species conservation of the p.170 glycine residue (star).

Cross-species conservation analysis by multiple alignment of the PPIB from human and 28 other mammal species showed that the p.170 glycine was highly conserved across species (Figure 5).

## 4. Discussion

In this study, by using a trio-WES study with comprehensive gene set analysis, a biparental-origin homozygous c.509G>A(p.G170D) mutation in *PPIB* was identified in two Taiwanese non-consanguineous families with fetal SD, supporting a diagnosis of OI IX (OMIM#259440).

SD is a group of disorders characterized by genetic and phenotypic complexity. OI is one of the vivid examples of genetic heterogeneity with different patterns of inheritance and wide variability of clinical severity. Despite 90% of the cases being found to harbor heterogeneous mutations in *COL1A1* and *COL1A2*, which cause the deficit in collagen type I biosynthesis, mutations in other genes causing OI are gradually being identified. The inheritance modes include AR and X-linked [22,23]. Currently, the classification system and diagnostic modality for OI has been modified on the basis of genetics and clinical features [24].

In our clinical setting for the genetic diagnosis of fetal SD, the first-line of gene testing was Sanger sequencing of a gene set including *FGFR3*, *COL1A1*, *COL1A2*, *SOX9*, and *DTDST*. However, with the advent of NGS and the introduction of WES, the cost of sequencing all the candidate genes by Sanger sequencing already exceeds that of WES. We thus shifted our second-line of gene testing from Sanger sequencing to WES, if negative findings were obtained in the first-line of gene test [25]. We preferred WES rather than the commercial panels as the second-line of gene testing because SD has been associated with more than 300 genes, but most commercial panels only include limited numbers of SD genes (e.g., 29 genes in CTGT: http://ctgt.net/panel/skeletal-dysplasia-core-extended-ngs-panel; 179 genes in FULGENT: https://fulgentgenetics.com/Skeletal-Dysplasias). Furthermore, for the large number of SD genes to be tested, the cost of WES (approximately USD 1000/test in Taiwan) is cheaper than most commercial panels (e.g., USD 1300/test for CTGT SD panel: 29 genes). Particularly, in order to cover diagnostic necessities and facilitate analytic processes, a comprehensive gene set that comprises almost all the reported 317 SD genes was included in our WES analysis pipeline before performing a genome-wide analysis with the aim to identify novel genes involved in the etiology of SD. To avoid overkill by using WES, we were very cautious in reporting any secondary findings unless in those genes strongly correlated with clinical phenotype.

OI IX (# OMIM 2590440), a subtype of OI corresponding to clinically severe type II/III of the Silence classification, is a monogenic disorder following AR inheritance. Most of the affected patients reported are from consanguineous couples [26,27,28]. The *PPIB* (located at chromosome 15q22.31) is considered as the causative gene of OI IX. This gene encodes for cyclophilin B (CYPB), a component of the collagen prolyl 3-hydroxylation complex in addition to cartilage-associated protein (CRTAP) and prolyl 3-hydroxylase (P3H1), is ubiquitously expressly throughout all types of tissues [29]. Proteins in this family have an enzymatic function named protein isomerase, which can catalyze the cis-/trans-conformation of the imidic peptide bond of proline residues to facilitate proper protein folding. This protein is mainly located at the nucleus, endoplasmic reticulum, and extracellular region according to Gene Ontology (GO) annotation. The primary function of PPIB on bone development is the catalytic activity of collagen fiber trimerization, which contributes to the pathogenesis of OI [26,27].

The amino acid p.G170 is not a reported position involved in the post-translational modification of the PPIB protein. However, according to the protein families database Pfam (https://pfam.xfam.org/), p.G170 resides in the cyclophilin type peptidyl-prolyl cis-trans isomerase domain (PF00160) [30], which is crucial for protein folding regulation of type I collagen [31]. As a result, the change of an amino acid without a side-chain (glycine, G) to a negatively charged aspartic acid (D) may introduce a structural and functional impact on PPIB.

Actually, the mutation c.509G>A in *PPIB* has been reported in one publication from China as a compound heterozygous mutation segregated in a Chinese OI IX family in Fukien [32]. The authors performed functional analysis on this specific allele and proved partial functioning in one carrier case [32]. Our results from the in silico prediction and cross-species conservation analysis also demonstrated a strong effect of the c.509G>A mutation in PPIB and cross-species conservation of the p.170 glycine residue, and thus provided additional evidence to support the deleterious nature of the mutation. Since the predominant Han Chinese population of Taiwan is originally from the same region of China, and the two Taiwanese families in our study were unrelated and non-consanguineous, it is plausible that the allele encompassing the mutation of c.509G>A is a result of the founder effect, a phenomenon we had observed in several other monogenic disorders such as aromatic I-amino acid decarboxylase deficiency [33] and AR renal tubular dysgenesis [34]. Among a whole genome sequencing project in the Taiwanese population organized by Taiwan Biobank on more than 1500 healthy individuals, a total of 1514 wild type (c.509G/c.509G) and three heterozygote carriers (c.509G/c.509A) on *PPIB* c.509 were recorded. The minor allele (c.509A) frequency in the Taiwanese population is estimated as 0.099%, which is slightly higher than the Asian population in the gnomAD-exon project (0.035%) and in the ExAC project (0.028%), supporting a candidate hotspot in the Taiwanese population. However, further large-scale study is needed to confirm this hypothesis by determining the frequency of this specific allele in the Han Chinese population of Taiwan.

## 5. Conclusions

WES study with comprehensive gene set analysis is helpful and feasible in the clinical diagnosis of SD diseases. Given the phenotypic heterogeneity and the huge number of genes involved (>300), we support the use of WES rather than gene panels for SD diagnosis when no specific disease suspicion emerges to infer candidate genes for analysis. The WES may be applied to some extent in the prenatal diagnosis of SD or other rare conditions, although more future research is needed. It is also noteworthy that the allele c.509G>A(p.G170D) mutation in *PPIB* reported in this study is a presumable hotspot in the Taiwanese population and deserves further studies.

## Figures and Tables

**Table 1:** Summary of the trio-based whole exome sequencing (trio-WES) results for the two families (Family 1 and Family 2) with fetal skeletal dysplasia (SD).

Case	Zygosity (P/M/F)	Genomic Coordinate ^†^	Gene	Associated Disease (Inheritance)	cDNA Change	Amino Acid Change	Type of Variation	dbSNP153	Allele Frequency	ClinVar
Family 1										
	homo/het/het	chr4 g.39270033A>G	*WDR19*	1.Cranioectodermal dysplasia 4; OMIM#614378 (AR) 2.Short-rib thoracic dysplasia 5; OMIM#614376 (AR)	NM_025132.4: c.3416A>G	p.Q1139R	Missense	rs75621037	0.00939	Uncertain significance
	homo/het/het	chr12 g.88058860A>G	*CEP290*	1.Bardet-Biedl syndrome 14; OMIM#615991 (AR) 2.Joubert syndrome 5; OMIM#610188 (AR) 3.Meckel syndrome 4; OMIM#611134 (AR) 4.Senior-Loken syndrome 6; OMIM#610189 (AR)	NM_025114.4: c.6806T>C	p.I2269T	Missense	rs200090371	0.00114	NA
	homo/het/het	chr15 g.64156744C>T	*PPIB*	OI type IX; OMIM#259440 (AR)	NM_000942.5: c.509G>A	p.G170D	Missense	rs199606428	0.00035	NA
Family 2										
	homo/het/het	chr15 g.64156744C>T	*PPIB*	OI type IX; OMIM#259440 (AR).	NM_000942.5: c.509G>A	p.G170D	Missense	rs199606428	0.00035	NA

**^†^** GRCh38 assembly; P, patient; M, mother; F, father. Homo, homozygous; het, heterozygous; AR, autosomal recessive; NA, not available; *WDR19*, WD repeat domain 19; *CEP290*, centrosomal protein 290; *PPIB*, peptidylprolyl isomerase B.

**Table 2:** *In silico* predictions of functional effects for the three genetic variations detected in this study.

	*WDR19* c.3416A>G(p.Q1139R)	*CEP290* c.6806T>C(p.I2269T)	*PPIB* c.509G>A(p.G170D)
Reference transcript	NM_025132.4	NM_025114.4	NM_000942.5
Prediction algorithm			
SIFT	Tolerated	Tolerated	Deleterious
Polyphen 2 HVar	Benign	Benign	Damaging
LRT	Deleterious	Neutral	Deleterious
Mutation Taster	Disease causing	Polymorphism	Disease causing
Mutation Assessor ^†^	Low	Low	High
FATHMM	Tolerated	Tolerated	Deleterious
PROVEAN	Neutral	Neutral	Deleterious
MetaSVM	Tolerated	Tolerated	Deleterious
MetaLR	Tolerated	Tolerated	Deleterious
FATHMM-MKL	Deleterious	Deleterious	Deleterious

^†^ High and low indicate the protein function was predicted as functional and non-functional, respectively.
